# Encapsulation, release and insecticidal activity of *Pongamia pinnata* (L.) seed oil

**DOI:** 10.1016/j.heliyon.2021.e06557

**Published:** 2021-03-24

**Authors:** Aloke Purkait, Ayan Mukherjee, Dipak Kumar Hazra, Kusal Roy, Pabitra Kumar Biswas, Ramen Kumar Kole

**Affiliations:** a Department of Soil Science and Agricultural Chemistry, Palli Siksha Bhavana (Institute of Agriculture), Visva - Bharati, Sriniketan, 731 236, Birbhum, West Bengal, India; b Department of Agricultural Chemicals, Bidhan Chandra Krishi Viswavidyalaya, Mohanpur, 741 252, Nadia, West Bengal, India; c Department of Agricultural Entomology, Bidhan Chandra Krishi Viswavidyalaya, Mohanpur, 741 252, Nadia, West Bengal, India

**Keywords:** *Pongamia* seed oil, Interfacial polymerization, Micro-encapsulation, GC-MS/MS quantification, Release kinetics, Insecticidal bio-assay

## Abstract

*Pongamia pinnata* (L.) seed oil is effective for its insecticidal and larvicidal activities. However, its low aqueous solubility, high photosensitivity, and high volatility restrict its application for the control of agricultural pests. Encapsulation can be an effective technique to overcome such hindrances. Therefore, *P. pinnata* oil (PO) was extracted from its seeds and analyzed for *karanjin* content (3.18%) by GC-MS/MS as the marker compound. Micro-encapsulation (MC) of PO was prepared by interfacial polymerization between isocyanates and polyamine and tested for insecticidal and larvicidal activities. Bioassay of the developed formulations was tested *in-vitro* against 2^nd^ instar larvae of *Bombyx mori* (Bivoltine hybrid) and *in-vivo* insecticidal bio-efficacy was tested against aubergine aphid (*Aphis gossypii* G.) and whitefly (*Bemisia tabaci* G.). Various properties of micro-capsules *viz.,* stability, size, oil content and release kinetics were examined. Average diameter of capsules (1 μm) with Zeta potential (-16 mV) was indicated by the Dynamic Light Scattering (DLS) instrument. Existence of PO in the microcapsules was confirmed by an optical microscopic study. Spectroscopic analysis revealed 87.4% of PO was encapsulated in polyurea shell. The shelf-life (*T*_*10*_), half-life (*T*_*50*_), and expiry-life (*T*_*90*_) of polyurea coated capsules were 11.4, 75.3 and 250.0 h, respectively. Polyurea coated PO capsule formulation showed evidence of *in-vitro* toxicity against 2^nd^ instar larvae of *B. mori* (*LC*_*50*_*=* 1.1%; *LC*_*90*_ = 5.9%). The PO formulation also exhibited 67.0–71.8% and 62.4–74.8% control of aphid and whitefly population in aubergine at 4.0% dose following 7–14 days after application. The study unveiled its significance in developing controlled release herbal formulations of *P. pinnata* as an alternative to harmful conventional synthetic insecticides for crop protection.

## 1 Introduction

Indiscriminate use of synthetic pesticides in crop protection is a major concern for environmental pollution affecting air, water, soil, human and animal health (Foong et al., 2020). Consequently, it is now an utmost necessity to find alternative means for sustainable crop protection with lower impacts on the environment (Nehra et al., 2020; Singh et al., 2019). Botanical pesticides can be promising alternatives to synthetic molecules, be cost-effective, IPM compatible and safe for the ecosystem and public health (Campos et al., 2018). The bioactive compounds produced in plants during secondary metabolism have been found with good insecticidal potency (Isman, 2017; Lengai et al., 2019). However, the rapid degradation of botanical extracts caused by volatilization and photo degradation of the active principles and their low solubility towards target surfaces are considered as disadvantages for utilizing these bio-inspired pesticides (Pant et al., 2016; da Maciel et al., 2019).

Encapsulation technique physically entraps the bioactive molecules into a protective matrix at the micro or nano range (Karadeniz et al., 2018; Ferreira and Nunes, 2019) and thereby, improve their delivery at the desired site and time at a specific rate (Bakry et al., 2016). Wall materials act as barriers to protect the core bioactive principles from degradation, evaporation and control diffusion to ensure sustained release (da Maciel et al., 2019). The usefulness of encapsulation technologies in botanicals in terms of increased solubility, protection against premature degradation, and sustained release of bioactive principles have also been reported (Bilenler et al., 2015; Oliveira et al., 2018).

*Pongamia pinnata* (L.) plant (Family: Fabaceae) is a medium-sized glabrous semi-evergreen tree growing in tropical regions (Malaikozhundan and Vinodhini, 2018). It is being cultivated in a large number of gardens and along the countless roads in India, attributed to having several medicinal and therapeutic properties (Rekha et al., 2020). The seeds of the plant containing *karanjin*, a furano-flavonoid compound have been reported to possess insecticidal activity (Raghav et al., 2019). A number of studies have reported the larvicidal activities of *P. pinnata* seed oil (PO) formulation (Pavela, 2012; Stepanycheva et al., 2014). In our previous communication, we also reported the preparation of emulsifiable concentrate (EC) formulation of PO with insecticidal properties (Purkait et al., 2019). The present study aimed to develop polyurea coated encapsulated PO formulation; their characterization in terms of thermodynamic stability and release kinetics; and evaluation of insecticidal bio-efficacy.

The biological efficacy of the developed controlled release formulation has been assessed against the important sucking pests (viz., aphid and whitefly), responsible for significant yield reduction of vegetable crops (Kodandaram et al., 2016; Chatterjee et al., 2018). *In-vitro* larvicidal bioassay of the PO formulation was also assessed against the insect *Bombyx mori* L. to understand its effectiveness against biting and chewing lepidopteran pests causing more than 40% yield loss in vegetable crops (Khan et al., 2020).

## 2 Materials and methods

### 2.1 Materials

Matured seeds of *P. pinnata* were procured from the local market at Mohanpur, District-Nadia, West Bengal, India. Karanjin standard (≥95.5% purity) was purchased from Chromadex, California, USA. N-hexane, Tween 20 (Polyoxyethylene sorbitan monolaurate), Tween 80 [Polyoxyethylene (20) sorbitan monooleate], Proclin 300, and Xanthan gum powder were procured from Sigma Aldrich, India. Sodium dodecyl benzene sulfonate (emulsifying agent) was obtained from Unitop Chemicals Pvt. Ltd., Gujarat, India. Toluene di-isocyanate (TDI), Ethylene diammine, and Diethylene triammine were purchased from Loba Chemicals, India and used as monomers for polyurea wall-forming materials. Reagents were used without further purification. Deionized water (milli-Q, Merck, Elix EE-22, Germany) was used in the formulation.

Primary secondary amine, PSA (Varian, Harbor City, CA); C18 (ODS - Octadecyl silane, Varian, Harbor City, CA); and Florisil (Acros Organics, Geel, Belgium) were used for extraction and clean-up of *P. pinnata* oil (PO) in GC-MS/MS analysis.

### 2.2 Preparation of *P. pinnata* seed oil

Seeds of *P. pinnata* were washed thoroughly to remove dust or other impurities, air-dried at room temperature and ground to a powder in an electrical grinder (Bajaj, Bravo DLX 500, India). The powdered seed (250 g) was extracted for 6 h as per the procedure described by Kage et al. (2016) using n-hexane (1 L) in the Soxhlet apparatus at 60 °C and the extract was filtered through Whatman filter paper No. (41) to obtain a clear filtrate. The solvent was evaporated from the filtrate to obtain the concentrated crude oil (70.92 g) at 40 °C using Rotary Vacuum Evaporator (R-300, Buchi, Switzerland) and stored at 4 °C for further use.

### 2.3 Karanjin quantification

#### 2.3.1 Preparation of standard solution

**Figure 1:**
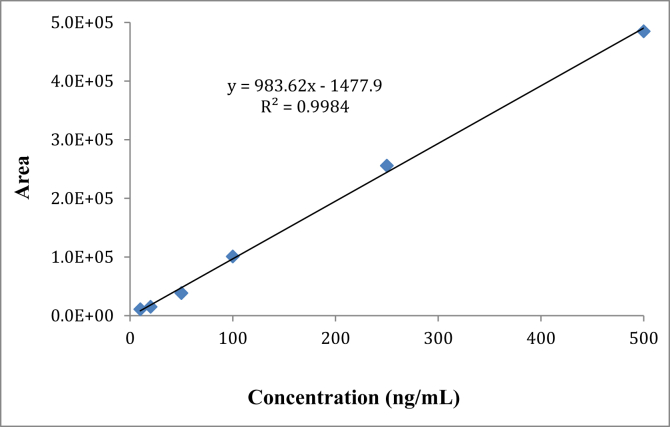
Calibration of *karanjin* by GC-MS/MS.

Accurately weighed 5 (±0.1) mg of *karanjin* (reference standard) was transferred to a calibrated volumetric flask (50 mL) and dissolved in ethyl acetate (50 mL). The stock solution thus prepared was placed in a refrigerator at 4 °C. A standard calibration curve (Figure 1; Y = 983.62X-1477.9; R^2^ = 0.9984) was plotted using intermediate standards concentrations (0.01, 0.02, 0.05, 0.10, 0.25 and 0.50 μg mL^−1^) from the above stock solution. The detection limit for *karanjin* was found to be 0.02 μg mL^−1^.

#### 2.3.2 Cleanup of *Pongamia* seed oil (PO)

The clean up procedure was adopted as described in our previous work (Purkait et al., 2019). The seed oil obtained from Soxhlet extraction was diluted in *n-*hexan*e* (HPLC grade) in 1:10 (w/w) ratio and subjected to centrifugation at 5000 rpm for 10 min at room temperature (~32 °C) followed by filtration of the supernatant through a nylon filter (0.2 μm). The filtrate was further diluted with *n-*hexane (HPLC grade) in 1:200 (w/w) ratio and transferred into a centrifuge tube (2 mL) containing PSA (50 mg), 25 mg C18 (ODS) and florisil (25 mg) for dispersive-solid phase extraction (d-SPE) to remove fatty acids (Grimalt and Dehouck, 2016) by centrifugation with Spinwin (Eltek-TC4100D, India) for 5 min at 5000 rpm. The clear aliquot obtained was filtered with a syringe filter (SGE Analytical Science Pvt. Ltd, India) fitted with 0.2 μm Nylon 66 membrane filter paper and transferred into vials for GC-MS/MS analysis.

#### 2.3.3 Recovery experiment

The diluted PO oil was spiked with standard solutions (0.02, 0.05 and 0.10 μg mL^−1^) of *karanjin* and subjected to d-SPE using PSA (50 mg), 25 mg C18 (ODS) and florisil (25 mg) as described under section 2.3.2. The supernatant thus obtained was filtered using syringe filter (0.2 μm membrane filter paper) and transferred to vials for estimation of *karanjin* in GC-MS/MS.

#### 2.3.4 Instrumentation

*Karanjin* was quantified by Gas Chromatography (GC: Trace 1300; Thermo Fisher Scientific, USA) equipped with a mass selective detector, triple quadruple analyzer (TSQ Duo 8000) and Triplus RSH Auto-sampler controlled by Xcalibur V3.1 software. Samples were injected under the following operating conditions as described in our previous works (Purkait et al., 2019; Ghada et al., 2017).

Helium was used as carrier gas at 1.0 mL min^−1^, in splitless mode. The solvent delay was 1 min and the injection volume was 1.0 μL. The mass spectrometric detector was operated in multiple reaction monitoring (MRM) mode with ionizing energy of 70 eV. The ion source temperature was 250 °C. The electron multiplier voltage (EM voltage) was maintained at 4000 V. The GC temperature program was started at 70 °C (1 min) then increased to 150 °C at a rate of 25 °*C min*^−1^, later increased up to 200 °C at 5 °*C min*^−1^ and finally the oven temperature was elevated up to 300 °C at 12 °*C min*^−1^ and hold for 6 min. The injector temperature was set at 290 °C.

**Figure 2:**
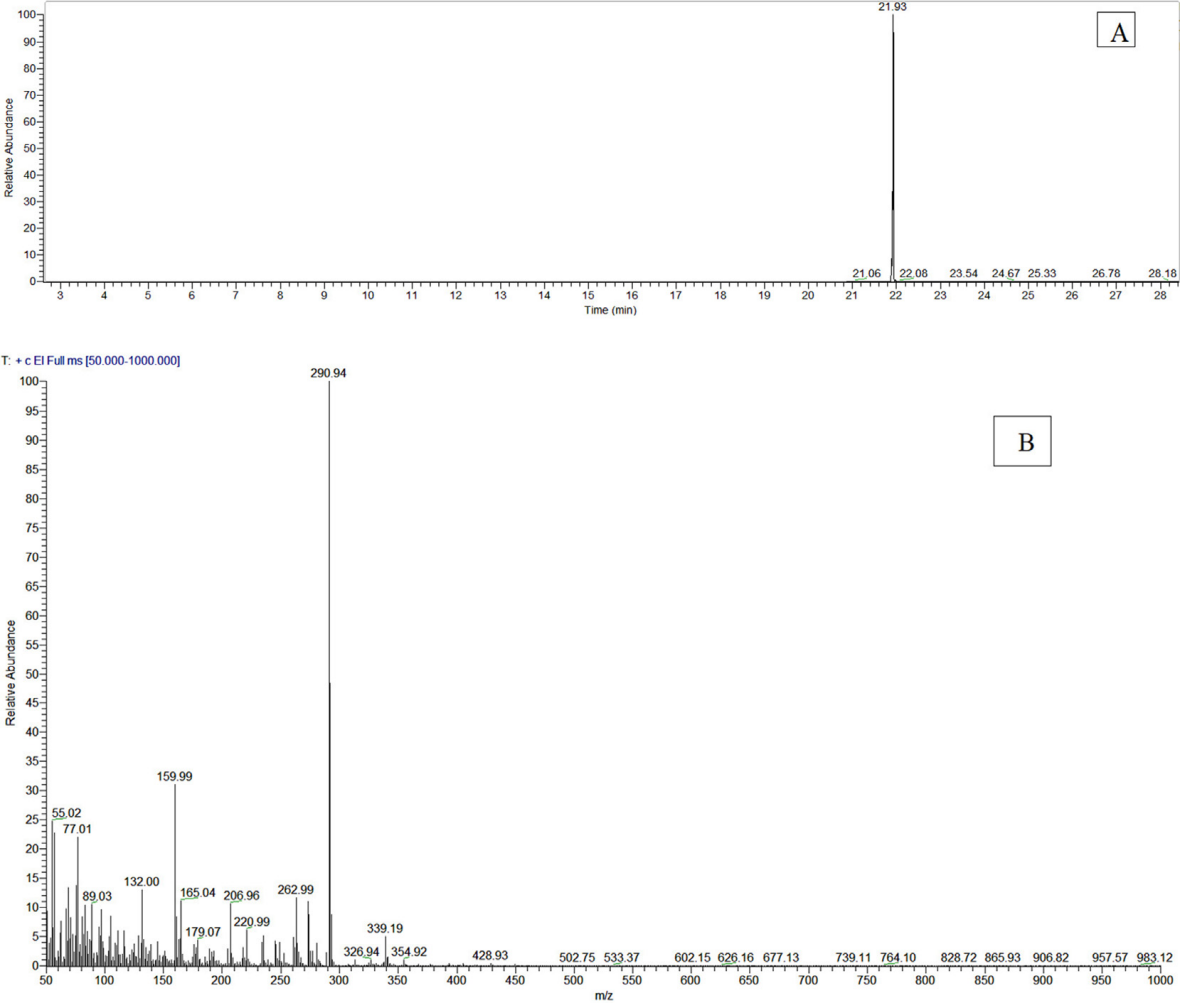
GC-MS/MS chromatogram of standard *karanjin* (A) and its mass fragmentation ions (B).

The MRM transition of *karanjin* selected is presented in Table 1. The retention time (RT) of the *karanjin* standard was recorded as 21.93 min (Figure 2A) and the corresponding mass spectrum is presented in Figure 2B.

**Table 1:** Multiple Reaction Monitoring (MRM) transition parameters and retention time of *karanjin*.

Analyte	Retention time (min)	Precursor ion (*m*/*z*)	Product ions (*m*/*z*)	Collision energy	Dwell time (sec)
*Karanjin*	21.93	291	179	18	0.147
			160	24	
			77	20

### 2.4 Encapsulation of seed oil

A two-step micro-encapsulation process was adopted from the method described by Podshivalov et al. (2013) with little modification. The formation of oil-in-water (o/w) emulsion (first step) was followed as elaborated in our previous work (Purkait et al., 2020) and the micro-encapsulation by interfacial polymerization under stirring conditions (second step) was as follows.

**Figure 3:**
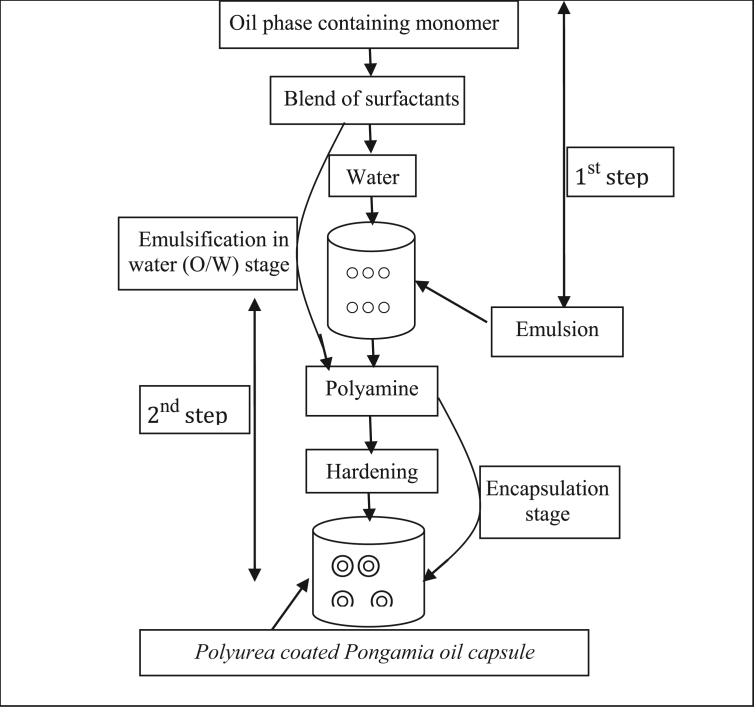
Schematic representation of Encapsulation by polymerization method.

I. Toluene-di-isocyanate monomer (0.1, 0.2, 0.6 and 1.0%, w/w) were solubilized in PO (2, 5, 7.5 and 10%, w/w) in a beaker (250 mL) followed by dropwise addition of blended surfactants (Sodium dodecylbenzene sulfonate, Tween 20 and Tween 80) over a magnetic stirrer (Remi, 2MLH, Maharashtra, India) at 60–100 rpm and 30–40 °C temperature. The mixture thus formed was added into deionized water at 30–40 °C under slow mixing (200–300 rpm) to produce a transparent faint yellow emulsion.
II. Different proportions of polymerizing agents (ethylene diammine and diethylene triammine) were mixed into deionized water and added drop-wise into stable emulsion formed in step I under slow mixing at 400–500 rpm to complete encapsulation of PO by interfacial polymerization at the oil-in-water interface (Figure 3).

Finally, previously prepared xanthene gum solution (0.2%) was added to the suspension to prevent the settling of microcapsules. Formaldehyde (0.5%) was used as a preservative to prevent microbial growth during storage.

### 2.5 Characterization of formulation

Unless otherwise referred, the physico-chemical properties of the encapsulated PO formulations were tested as per the Collaborative International Pesticides Analytical Council (CIPAC) and Indian Standard (IS) specifications (BIS, 1997). All the experiments were done in triplicate.

#### 2.5.1 pH measurement

The pH value of PO formulation was measured by immersing the electrode directly into the emulsion using a pH meter (Systronics, Model 335, Gujarat, India) at 25 ± 1 °C (CIPAC MT75.3, 2000). Before recording the value, pH meter was calibrated using pH 4.0, 7.0 and 9.2 buffer solutions (Merck).

#### 2.5.2 Thermodynamic stability study

For stability study, formulated PO was subjected to different tests viz., centrifugation, heating-cooling cycle and freeze-thaw stress. Initially, the formulations, which passed the thermodynamic stress tests, were considered for further characterization.

##### 2.5.2.1 Centrifugation

Encapsulated PO formulations were centrifuged (Eltek, TC-4100D, India) at 3000 rpm for 30 min and observed for any phase separation. Stable samples without separation were analyzed for heating-cooling cycles and freeze-thaw stress.

##### 2.5.2.2 Heating-cooling cycle and freeze-thaw stress

Heating-cooling cycle was performed by placing PO formulation at 4 °C and 40 °C, alternating at each temperature for 48 h. The cycle was repeated thrice. For freeze-thaw stress, the botanical formulation was kept alternatively at 25 °C and -21 °C for 48 h twice. All tests were carried out as per the standard methods outlined in the CIPAC Handbook (CIPAC MT46.3, 1995).

#### 2.5.3 Microcapsule's morphology

The morphology of the synthesized microcapsules was examined by Optical Microscope (Nikon, CIS, Tokyo, Japan) equipped with the image capturing (Nikon, DS-Ri2) and image analyzing device (NIS-Elements, BR 4.30.00). An optimized image of microcapsules was captured by exposure adjustments.

#### 2.5.4 Droplet size distribution

The mean droplet size and zeta potential of bio-formulation were measured by Zetasizer (Nano-ZS90, Malvern, Worcestershire, UK) based on the principles of Dynamic Light Scattering (DLS).

### 2.6 Determination of encapsulation efficiency

**Figure 4:**
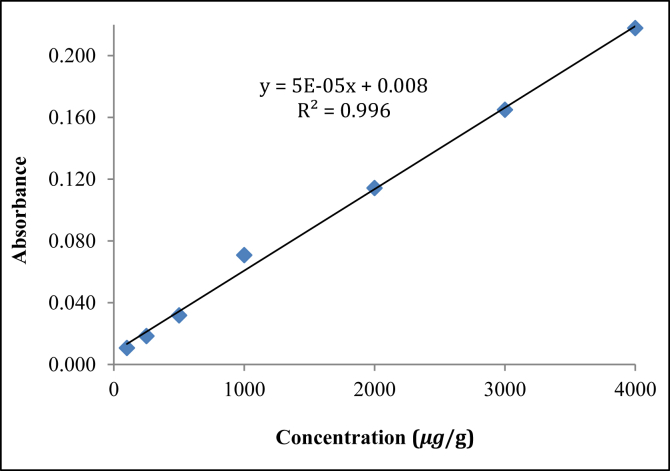
Spectrophotometric calibration of *Pongamia* oil.

The encapsulation efficiency (EE) was determined by dissolving free oil (PO) in the developed formulation in *n-*hexane as the polyurea capsule containing PO is insoluble in *n-*hexane (Ghahfarokhi et al., 2016). Encapsulated formulation (4 mL) was mixed in *n-*hexane: deionized water (1:3 v/v) mixture (266.7 mL) at 32 °C by vortex for 1 min followed by centrifugation. The upper *n-*hexane layer was collected and filtered through 0.2 μm nylon filter paper for quantification by Spectrophotometer (Varian, EL 06053741, USA) at 259 nm. A standard calibration curve (Figure 4) was prepared using different concentrations (100–4000) μg mL^−1^ of oil. The EE was calculated using the following Eq. (1).

(1) $$Encapsulation\;efficiency\left(\%\right)=\frac{Total\;amount\;of\;oil\;in\;the\;formulation-Free\;oil}{Total\;amount\;of\;oil\;in\;the\;formulation}X\;100$$

#### 2.6.1 In-vitro release of encapsulated PO

Release of PO from the suspended microcapsules in the formulation was determined following the modified method of Marcela et al. (2015). Time-dependent release of PO formulation (4 mL) was studied in the mixture of *n-*hexane and deionized water (1:3 v/v). The formulation mixture was shaken in Incubator Shaker (Zhcheng, ZHWY-200D, Shanghai, China) @ 300 rpm at 32 °C. The release was monitored at different time intervals (0–48 h). An aliquot (3 mL) was withdrawn and replaced with a freshly mixed solution, centrifuged, filtered through 0.2 μm nylon filter and quantified spectrophotometrically. All the measurements were made in triplicate. The release of PO was quantified using equation II as follows.

(2) ReleaseofPongamiaoil(%)=ReleasedoilTotaloilintheformulation×100

#### 2.6.2 Kinetics of PO release

The logarithmic values of the released PO concentration (C X 1000) were graphically plotted against different times (t) to produce a straight line equation (III) indicating the first-order nature of PO release from polyurea microcapsules.

(3) $$\text{log}\;{\text{C}}_{t}=\text{log}\;{\text{C}}_{0}-\text{K}/2.303\;\text{X}\;\text{t}$$

where,

C_t_ = Concentration of PO at a particular time, t
C_0_ = Initial concentration of PO in the formulation
K= Rate constant for PO release from the formulation

Half-life (*T*_*1/2*_) or *T*_*50*_ (i.e. time required for 50% release) of PO was calculated by replacing C_t_ and t with 0.5C_0_ and *T*_*50*_, respectively in the above equation (III) to obtain equation (IV).

(4) $$T_{50}=0.693\text{/K}$$

The shelf-life (*T*_*10*_) (time required for 10% release of PO) was calculated by replacing C_t_ and t with 0.9C_0_ and *T*_*10*_; whereas the expiry life, *T*_*90*_ (time required for 90% release of PO) was calculated by replacing C_t_ and t with 0.1C_0_ and *T*_*90*_ in equation III to obtain equation V and VII respectively (Agrahari et al., 2014).

(5) $$T_{10}=0.105\text{/K}$$

(6) $$T_{90}=2.303\text{/K}$$

### 2.7 Bio-efficacy of encapsulated PO formulation

#### 2.7.1 In-vitro larvicidal activity

*In-vitro* bio-efficacy of the bio-inspired formulation was assayed against 2^nd^ instar larvae of *Bombyx mori* (Bivoltine hybrid) following leaf dip bioassay technique (Paramasivam and Selvi, 2017) at Sericulture Unit, Dept. of Agricultural Entomology, Bidhan Chandra Krishi Viswavidyalaya (BCKV) at Mohanpur, Dist. Nadia, West Bengal (India) during the month of February–March, 2018. Eggs were collected from Central Sericulture Research & Training Institute, Berhampore, Dist. Murshidabad, West Bengal. Fresh mulberry (Variety: S 1635) leaves (*Morus alba* L.) were collected from the fields and washed under tap water. Treatments (T_1_-T_5_) of PO formulation (0.2, 0.4, 1.0, 2.0 and 4.0%) prepared in distilled water along with distilled water as untreated control (T_6_) were tested on freshly hatched 2^nd^ instar larvae (40 nos.) under controlled laboratory conditions (Temp. = 27 ± 2 °C; Relative Humidity, RH = 80 ± 5%; Exposure to light and dark, L:D = 16:8 h). The experiment was conducted in a Completely Randomized Design (CRD) with four replications for each treatment.

Chopped leaves were dipped into the prepared concentration of PO formulation for 9–10 s and air-dried under the shade at room temperature (27 ± 2 °C) for 20 min. Treated bits of leaf were placed in a container and the larvae were released. A small piece of cotton cloth was fastened by a rubber band on the mouth of the container to prevent the escape of the larvae. In an untreated control, larvae were fed with distilled water treated and shade dried mulberry leaf bits only. Mortality of larvae was recorded after 24, 48, 72 and 96 h of exposure.

Statistical analysis was carried out in CRD after the necessary transformation of the data and subjected to Duncan's Multiple Range Test (DMRT) using SPSS software (version 24). Abbott (1925) was used to correct the mortality considering the mortality that occurred in the untreated check. Percentage mortality (means ± SE) of larvae was recorded against different doses of the formulation. The *LC*_*50*_ and *LC*_*90*_ values of the PO formulation were calculated from the logarithm of each concentration and corresponding probit value to each inhibition percentage (Finney, 1971).

#### 2.7.2 In-vivo insecticidal bio-efficacy

A field experiment was conducted to evaluate the efficacy of the developed PO formulation against aphid and whitefly of brinjal (cv: *Pusa Purple Long*) in the District Seed Farm (AB - Block), BCKV, Kalyani during August–September, 2018. The experiment was conducted in a Randomized Block Design (RBD) with a total of five (T_1_-T_5_) treatments with four replications for each treatment [PO formulations @ 1.0% (T_1_) and 4.0% (T_2_); two recommended insecticides in India (CIBRC, 2018) *viz.,* Lambda (λ) cyhalothrin 5% EC @ 0.05% (T_3_) and Cypermethrin 10% EC @ 0.1% (T_4_) along with tap water as untreated control (T_5_)]. All the treatments were applied twice at 14 days intervals as foliar spray during the fruiting stage of the crop using a pneumatic knap-sack sprayer (ASPEE, Kolkata, India) fitted with a hollow-cone nozzle. The data on targeted insect-pests were recorded from five randomly selected and tagged plants of each plot. Observations on the total number of aphids and whiteflies were noted from three leaves (one each at the top, middle and bottom) per plant. First count (pre-treatment, PT) of insect population before the first spray and post-treatment counts after 1, 3, 5, 7, 10 and 14 days of spray were recorded from the previously tagged plants. Reduction of aphid or whitefly population for each insecticidal treatments were corrected using Henderson and Tilton's formula (Henderson and Tilton, 1955) as shown below;

$$\text{\%}\;\text{Reduction}=1-\frac{\text{n}\;\text{in}\;\text{Co}\;\text{before}\;\text{treatment}\;x\;\text{n}\;\text{in}\;\text{T}\;\text{after}\;\text{treatment}}{\text{n}\;\text{in}\;\text{Co}\;\text{after}\;\text{treatment}\;x\;\text{n}\;\text{in}\;\text{T}\;\text{before}\;\text{treatment}}\times 100$$

where, n = Insect population; T = Treated, Co = Control.

Statistical analysis was carried out for working out the ANOVA after making a suitable transformation of the data followed by comparison of means between the treatments using Duncan's Multiple Range Test at p < 0.05 (Gomez and Gomez, 1984).

## 3 Results and discussion

In the first step of encapsulation (oil-in-water emulsion), we used a low energy emulsification method without using a sonicator or high-speed homogenizer. *P. pinnnata* seed oil (2–10%) was subjected to encapsulation using emulsifier blends, monomers (isocyanate, polyamine), glycerol (2%), or propylene glycol (4%) (anti-freezer) and remnant water. Oil-in-water (O/W) emulsion with 2–5% of PO visually resulted in the lowest turbidity and passed the thermodynamic stability test successfully. Emulsion produced with 7.5% PO was unstable with phase separation during thermodynamic stability study. PO emulsion (5%) was further polymerized with different concentrations (Table 2) of polyamine (0.2–0.4%) and isocyanate (0.1–1%). Among the five formulations developed, MC_1_ and MC_4_ were found stable of which MC_1_ was chosen for further physico-chemical characterizations it required the least amount of surfactant.

**Table 2:** Process optimization for micro-encapsulation of *Pongamia* Oil (PO).

Formulation Code	Composition (%w/w)				Appearance	pH	Thermodynamic stability study		
	PO	TDI	Blend surfactant	Polyamines (1:1)			Centrifugation	Heating-cooling cycle	Freeze-thaw stress
MC_1_	5.0	0.1	18	0.2	Faint yellow viscous	6.68	Pass	Pass	Pass
MC_2_	5.0	0.4	25	0.2	Faint yellow viscous	6.60	Fail	Fail	Fail
MC_3_	5.0	0.6	22	0.3	Faint yellow viscous	6.65	Pass	Pass	Fail
MC_4_	5.0	0.4	22	0.4	Faint yellow viscous	6.76	Pass	Pass	Pass
MC_5_	5.0	1.0	25	0.4	Phase separation	6.78	Fail	Fail	Fail

PO = *Pongamia oil*; TDI = Toluene-di-isocyanate; Blend surfactant = Tween 20, Tween 80 and sodium dodecyl benzene sulfonate (1:2 to 1:1); Polyamines = Diethyl triamine and ethylene diamine.

### 3.1 Formation and stability of polyurea-coated capsules

The polymer-based coating material (polyurea) was formed via interfacial polymerization of monomers (diisocyanate and polyamine) at the oil-in-water emulsion interface (Perignon et al., 2014). The polymerization technique was optimized with the variation of PO, TDI and polyamine concentrations to ensure the stability of the formulation. Microcapsules of the pesticide ethion were synthesized by Moghbeli et al. (2011) via interfacial polycondensation of 2,4-tolylene diisocyanate and ethylene diamine.

The concentration of monomer was a decisive factor for controlling the completion of the polymerization process (hardening time). Hardening time of 3–4 days was reported when more than 2.0% of polyamine was used (Perignon et al., 2014). The polyamine promotes catalytic action between amine and isocyanate to produce a polyurea wall by donating the electrons of the nitrogen atom of an amine group to the positively charged carbon atom of the isocyanate (Sharmin and Zafar, 2012). Coalescent and aggregation of capsules in the present experiment were observed when the PO concentration was increased by more than 10%. We observed that isocyanate and polyamine ratio is the primary factor for the stability of the encapsulation formulation. Whereas, the addition of an excess surfactant (>30%) reduced the interfacial tension to decrease microcapsule size which was also affected by higher isocyanate (>1.0%) concentrations (Chao, 1993). However, the selected formulation, MC_1_ with the lowest surfactant content (18%) was stabilized using optimum isocyanate and amines concentration to reduce microcapsules sizes. The phase separation in MC_5_ formulation may be due to increased capsules size with increasing TDI concentration. The aqueous-based encapsulated PO formulations prepared from the biodegradable ingredients (*viz.,* Tween 20, Tween 80, SDBS, xanthenes gum, etc.) were stable up to twelve months.

### 3.2 Physico-chemical properties of PO formulation

The physicochemical characterization of the micro-capsule formulation was considered as different parameters. The polyurea coating matrix with PO was stable at the pH range of 6.6–6.8 (Table 2). No phase separation was observed after centrifugation of capsulated PO and also found thermodynamically stable. The micro-capsule formulation (MC_1_) with transparent faint yellow appearance turned translucent after hardening (2 h) and was found stable in heating-cooling cycle and freeze-thaw stress.

#### 3.2.1 Droplet size distribution

**Figure 5:**
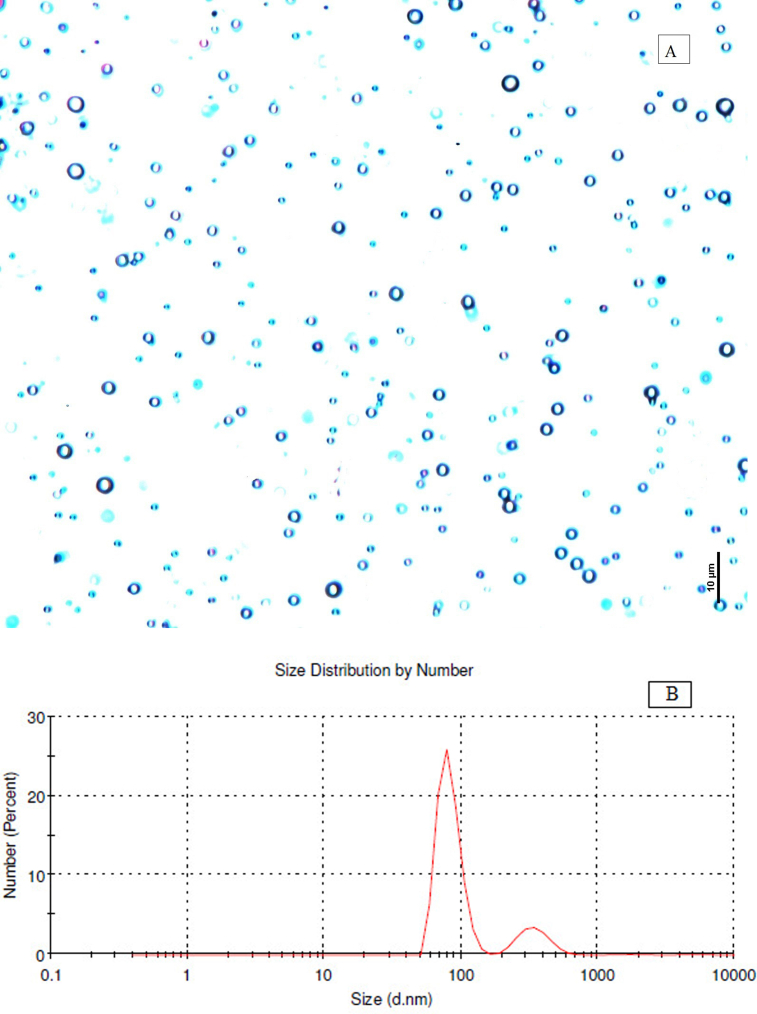
Formation of *Pongamia* oil Capsules (MC_1_) as indicated by: (A) Optical microscopy; and (B) DLS analysis.

DLS analysis revealed that 25% of capsules formed with hydrodynamic diameter of ≤100 nm (Figure 5B). The average size (1655 nm) of the microcapsules formed appeared to be much smaller than the earlier reports (Beestman and Deming, 1983; Kamble et al., 2018; Yadav et al., 1990). The initial formation of finer emulsion promoted the formation of smaller sized capsules (Yuan et al., 2019). No significant change in average capsule size distribution was observed by further DLS study of the two-month-old formulation. Maximum stability of the emulsion was observed due to the high zeta potential value (16.5 mV). The study proved that smaller sized capsules were formed in the optimum concentration of isocyanates and polyamines (1:1 v/v).

Optical microscopic analysis portrayed that all the droplets of PO have been individually encapsulated as spherical capsules with consistent size distribution as bimodal distribution (Figure 5A). Similar morphological characteristics have also been reported in the microencapsulated oils from *Annona squamosa* (da Maciel et al., 2019) and Jasmine (Teeka et al., 2014).

### 3.3 Encapsulation efficiency (EE) and release of PO

Spectroscopic analysis of PO encapsulation revealed that 87.4% of the oil was encapsulated (EE) by the only monomer isocyanates (0.1%) compared to the earlier report (EE = 94–98.4%) with a high amount (2–7%) of monomers (Marcela et al., 2015). Optimum level of encapsulation of oil inside the polyurea shell was successful under medium mixing at 400–500 rpm rather than lower (50–100 rpm) or higher (>2000 rpm) stirring speed. Microcapsule formation depends on pH of the medium, temperature, type and amount of emulsifier, co-emulsifier, matrix-forming agent, stirring speed, organic phase, etc. (Ollier and Alvarez, 2017).

**Figure 6:**
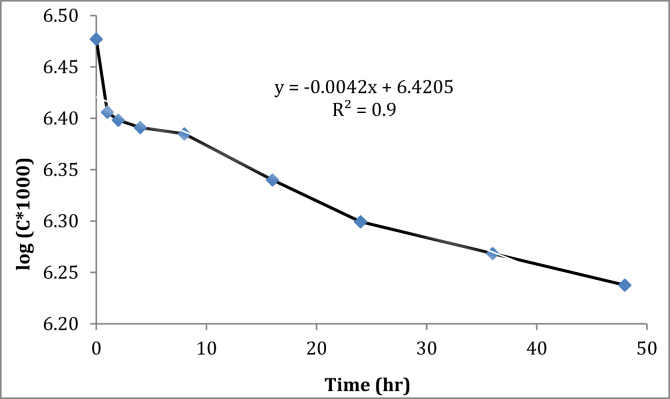
*In-vitro Pongamia* oil release kinetics from polyurea capsules.

The release of oil from the encapsulated polyurea was conducted under controlled laboratory conditions at room temperature (32 °C). The release assays provided the information about the release profiles of PO with time, either free or encapsulated in the polymer microcapsules. Release kinetic was monitored to measure the release rate of the encapsulated oil (Figure 6) as a function of content and the concentration of TDI and polyamines. The probable release mechanisms of the oil from microcapsules may include mechanical, chemical, or, thermal stimuli as well as diffusion (Ghayempour and Montazer, 2016).

The shelf-life (*T*_*10*_), half-life (*T*_*50*_) and expiry-life (*T*_*90*_) of PO from the polyurea coated capsules were mathematically calculated as 11.4, 75.3 and 250 h, respectively. Slower and more sustained release of PO was observed due to the smaller polyurea coated microcapsules. Hence, the coated capsules were more consistent than their larger counter parts (Yang et al., 2008).

Polyurea is an elastomer membrane, insoluble in water and deteriorates slowly (Marcela et al., 2015). The release of PO might have been facilitated through diffusion or after bursting of the wall membrane. Sustained release of PO (*T*_*90*_ = 250 h) suggests that the coating material polyuria was well suited for the encapsulation of hydrophobic compounds like PO. The release performance is much more superior to the earlier findings of 4–12 days (Pavela and Herda, 2007). Sansukcharearnpon et al. (2010) found that most patented and commercialized agrochemical formulations are based on micro and nano-encapsulation for improved persistence and reduced active ingredient losses.

### 3.4 Bio-efficacy of encapsulated *Pongamia* seed oil (PO)

#### 3.4.1 In-vitro larvicidal efficacy against *Bombyx mori*

The results of *in-vitro* bio-efficacy studies of the selected *Pongamia* capsule formulation (MC_1_) against *B. mori* larvae are presented in Table 3. More than 50% mortality of the test insect was observed at 1% dose (T_3_) after 72 h of treatment (Table 3). However, the higher doses (T_4_ and T_5_) of the MC_1_ fetched the same mortality alike T_3_ within 48 h. Much lower larvicidal mortality was recorded (≈10–20%) at the lower doses (T_1_ and T_2_) even after 96 h of application. The calculated lethal concentrations (*LC*_*50*_ and *LC*_*90*_) were found to be 1.06% and 5.87%, respectively after 96 h of insecticidal treatment.

**Table 3:** Larvicidal activity of polyurea encapsulated *P. pinnata* oil (PO) formulation (MC_1_) against *Bombyx mori*.

Treatment/Doses (% PO in water)	Mean mortality∗(%) of *B. mori* larvae after different time (hr) after application			
	24 h (±SE)	48 h (±SE)	72 h (±SE)	96 h (±SE)
0.2 **(**T_1_**)**	0^a^	0^a^	7.4^e^ ± 0.18	9.3^e^ ± 0.26
0.4 **(**T_2_**)**	0_a_	6.1^e^ ± 0.09	16.7^d^ ± 0.09	21.8^d^ ± 0.15
1.0 **(**T_3_**)**	5.2^d^ ± 0.21	32.5^d^ ± 0.18	54.3^c^ ± 0.24	56.5^c^ ± 0.23
2.0 **(**T_4_**)**	8.7^c^ ± 0.15	58.3^c^ ± 0.22	62.5^b^ ± 0.20	72.4^b^ ± 0.24
4.0 **(**T_5_**)**	10.3^b^ ± 0.24	69.4^b^ ± 0.15	75.2^a^ ± 0.17	77.6^a^ ± 0.17
Control (water) **(**T_6_**)**	0^a^	0.33^f^ ± 0.02	0.48^f^ ± 0.01	0.82^f^ ± 0.02
***LC***_***50***_	**0.01%**	**0.09%**	**0.08%**	**1.06%**
***LC***_***9*0**_	**0.06%**	**0.44%**	**0.38%**	**5.87%**

∗Means with the same letter are not significantly different according to Duncun Test (p < 0.05); Values are average of four replicates; SE = Standard error.

**Figure 7:**
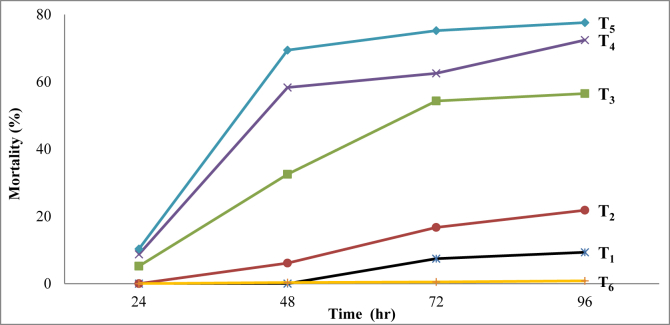
*In-vitro* mortality of *B. mori* larvae by *P. pinnata* oil (PO) formulation (MC1). [PO @ T_1_ = 0.2%; T_2_ = 0.4%; T_3_ = 1.0%; T_4_ = 2.0%; T_5_ = 4.0%; T_6_ = Control (distilled water)].

The larvicidal mortality produced by different doses of PO formulation (MC1) is also presented in Figure 7. The slope of the mortality-time curve represents the variability in response to treatments against the insect population (Figure 7). The larvicidal activity significantly (p < 0.05) increased with the increase in application doses from 0.20% to 4.0% (T_1_-T_5_). T_5_ (@ 4.0%) exhibited the highest larvicidal activity (77.6%) followed by T_4_ (72.4%) and T_3_ (56.5%) after 96 h of application (Table 3). The observed activity was comparable to the earlier report of 65% mortality of crude *n-*hexane extract of *P. pinnata* at 1% dose (Reena and Singh, 2007).

#### 3.4.2 In-vivo insecticidal activity

The results pertaining to the control of aphids and whiteflies following application of the botanical pesticide on brinjal under field conditions are presented in Tables 4 and 5, respectively. The population of aphids gradually decreased (p < 0.05) up to 10 days after application @ 1% (T_1_) and 4% (T_2_) doses of the developed bio-formulation (MC_1_). Maximum reduction (88–95%) of aphids was observed in the chemical insecticides *viz.,* Lambda (λ) cyhalothrin (T_3_) and Cypermethrin (T_4_) recommended for managing aphids in brinjal (CIBRC, 2018). The maximum bio-efficacy of MC_1_ @ 4% (T_2_) in terms of reduction of aphid population (71.8%) after 10 days of the foliar application was better compared to the conventional 40% EC (emulsifiable concentrate) formulation of PO (68.4%) as reported in our previous publication (Purkait et al., 2019).

**Table 4:** Effect of *Pongamia* oil formulation (MC_1_) on aphid infestation in brinjal under field condition.

Insecticide Formulation	Treatment/Doses (%)	Mean no. of aphids per 3 leaves per plant at pre-treatment (PT) and at different days (D) after treatment							% reduction of aphids after
		PT	1D	3D	5D	7D	10D	14D	7D 10D 14D
*P. pinnata* (5 MC_1_)	1.00 (T_1_)	19.16 (4.43)	17.13 (4.20)	15.89 (4.05)	13.62 (3.76)	13.14 (3.69)	11.81 (3.51)	13.64 (3.76)	43.68 52.58 52.14
*P. pinnata* (5 MC_1_)	4.00 (T_2_)	19.19 (4.44)	15.80 (4.04)	13.96 (3.80)	10.87 (3.37)	7.69 (2.86)	7.03 (2.74)	8.20 (2.95)	67.05 71.77 71.22
λ-Cyhalothrin (5 EC)	0.05 (T_3_)	19.17 (4.44)	6.07 (2.55)	5.04 (2.34)	3.71 (2.05)	1.11 (1.27)	1.24 (1.31)	2.24 (1.65)	95.26 95.03 92.15
Cypermethrin (10 EC)	0.10 (T_4_)	19.18 (4.44)	4.14 (2.15)	3.03 (1.87)	1.94 (1.55)	1.80 (1.51)	1.95 (1.56)	3.38 (1.94)	92.27 92.17 88.39
Control (Water)	- (T_5_)	19.15 (4.43)	19.17 (4.43)	20.56 (4.58)	21.06 (4.64)	23.33 (4.88)	24.90 (5.04)	28.50 (5.38)	0 0 0
**S.Em±**	**-**	**0.04**	**0.10**	**0.09**	**0.08**	**0.08**	**0.07**	**0.10**	**0.53 0.51 0.80**
**CD (p = 0.05)**	**-**	**0.13 (S)**	**0.30**	**0.28**	**0.22**	**0.23**	**0.19**	**0.31**	**1.76 1.70 2.67**

Values in parentheses are square root transformed; 5MC_1_: Micro-capsules of *P. pinnata* seed extract (5%); PT: Pre-treatment; SEm = Standard error of mean; CD = Critical difference; S = Significant difference.

**Table 5:** Effect of *Pongamia* oil formulation (MC_1_) on whiteflies infestation in brinjal under field condition.

Insecticide Formulation	Treatment/Doses (%)	Mean no. of whiteflies per 3 leaves per plant at pre-treatment (PT) and at different days (D) after treatment							% reduction of whiteflies after
		PT	1D	3D	5D	7D	10D	14D	7D 10D 14D
*P. pinnata* (5 MC_1_)	1.00 (T_1_)	1.10 (1.26)	1.06 (1.25)	1.01 (1.23)	0.91 (1.19)	0.86 (1.17)	1.27 (1.33)	1.47 (1.40)	55.73 44.23 43.30
*P. pinnata* (5 MC_1_)	4.00 (T_2_)	1.04 (1.24)	0.92 (1.19)	0.85 (1.16)	0.76 (1.12)	0.69 (1.09)	0.58 (1.04)	0.97 (1.21)	64.79 74.74 62.37
λ-Cyhalothrin (5 EC)	0.05 (T_3_)	0.98 (1.22)	0.40 (0.95)	0.29 (0.89)	0.27 (0.88)	0.07 (0.76)	0.06 (0.75)	0.12 (0.79)	96.24 97.52 95.23
Cypermethrin (10 EC)	0.10 (T_4_)	1.06 (1.25)	0.33 (0.91)	0.21 (0.84)	0.18 (0.82)	0.15 (0.80)	0.12 (0.79)	0.23 (0.85)	92.48 94.89 91.24
Control (Water)	- (T_5_)	1.14 (1.28)	1.23 (1.31)	1.26 (1.32)	1.52 (1.42)	1.95 (1.56)	2.28 (1.66)	2.59 (1.75)	0 0 0
**SEm±**	**-**	**0.05**	**0.06**	**0.06**	**0.06**	**0.05**	**0.05**	**0.05**	**0.27 0.34 0.35**
**CD (p = 0.05)**	**-**	**0.15 (S)**	**0.16**	**0.17**	**0.16**	**0.15**	**0.15**	**0.16**	**0.88 1.12 1.17**

Values in parentheses are square root transformed; 5MC_1_: Micro-capsules of *P. pinnata* seed extract (5%); PT: Pre-treatment; SEm = Standard error of mean; CD = Critical difference; S = Significant difference.

The population of whitefly gradually decreased (Table 5) over time after application of *P. pinnata* formulations (T_1_ and T_2_). Maximum reduction (94.9–97.5%) was noticed in the chemical insecticides *viz.,* Lambda (λ) cyhalothrin (T_3_) and Cypermethrin (T_4_) recommended for managing whitefly in brinjal (CIBRC, 2018). The encapsulated oil formulation (MC_1_) @ 4% (T_2_) exhibited 74.7% reduction of whitefly infestation at 10 days after spray (Table 5) which was superior to the conventional 40 EC (62.2%) as reported earlier (Purkait et al., 2019). However, Stepanycheva et al. (2014) reported 96% control of green peach aphid (LC50: 0.36%) at an application of 3% emulsion of *P. pinnata*. The variation in mortality of the targeted pest might be due to the content of active principles in the formulated botanical products (Dougoud et al., 2019). Therefore, the major bioactive compound (*karanjin)* present in *P. pinnata* oil was also estimated.

### 3.5 Karanjin estimation

**Figure 8:**
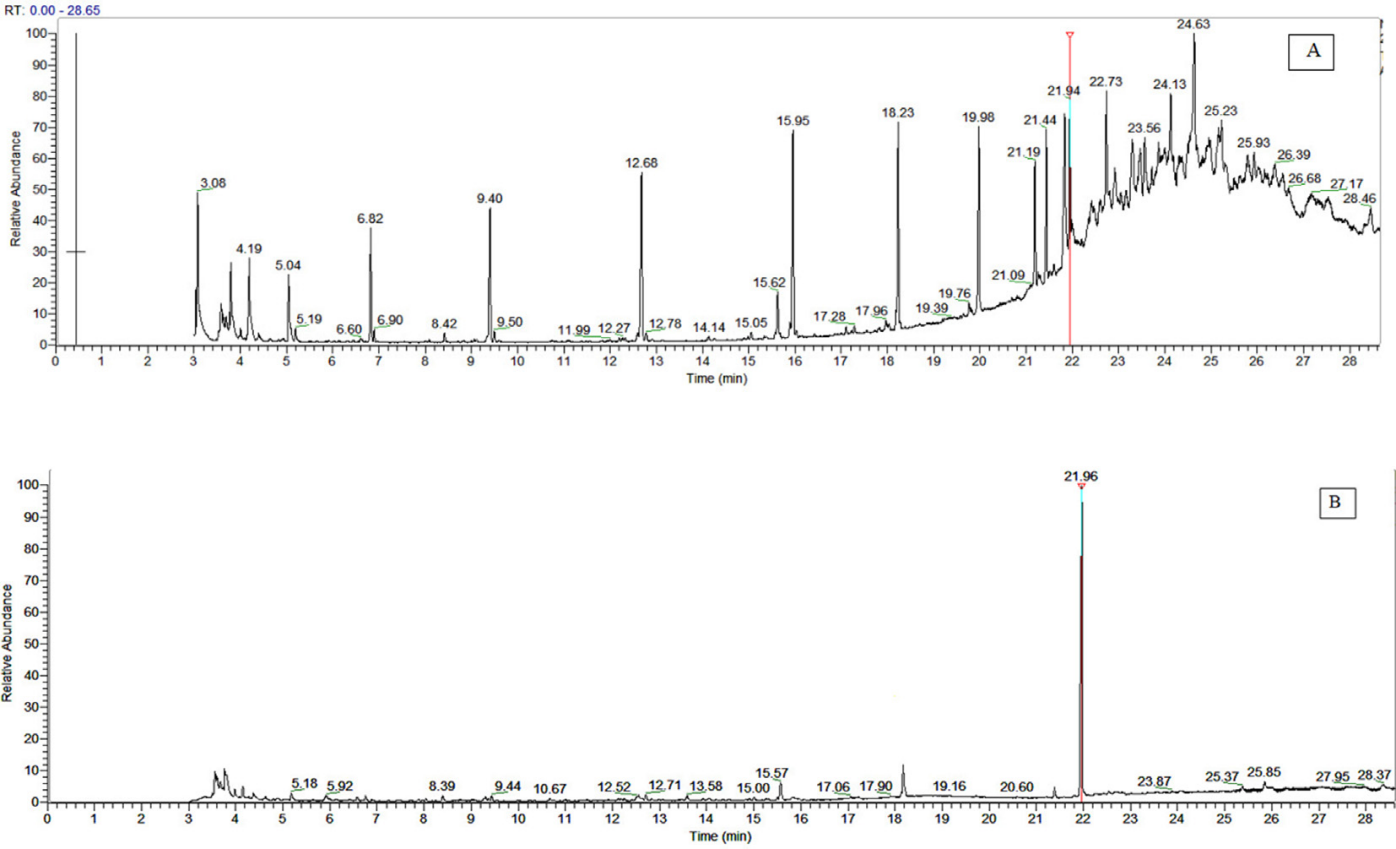
GC-MS chromatogram of *P. pinnata* oil by: (A) Total Ion Monitoring (TIM); and (B) Selected Ion Monitoring (SIM).

The method for estimation of *karanjin* by GC-MS/MS was standardized with limit of detection (LOD = 0.02 μg mL^−1^) and limit of quantification (LOQ = 0.05 μg g^−1^). The average recovery percentage of *karanjin* was 94.44% (Table 6). GC-MS analysis of *Pongamia* seed oil showed the presence of *karanjin* (RT = 21.94 min) along with a number of other phytochemical constituents (Figure 8A). *Karanjin* content *in* seed oil (3.18%) was determined by comparing the peak area of *karanjin in* seed oil with the standard (Figures 2A and 8B). *Karanjin* content in petroleum ether extract of *P. pinnata* seed was also found in the range of 3.20–5.43% (Pavithra et al., 2012; Vismaya et al., 2010). Gore and Satyamoorthy (2000) also estimated *karanjin* content in *Pongamia* seed oil in the range of 1.1–4.5%.

**Table 6:** Results of method validation of *karanjin* for clean-up procedure.

Level of *karanjin* fortified (μg/g)	Amount recovered (μg/g)			Average Amount recovered (μg/g) ± s.d.	Recovery (%)	Mean Recovery (%)
	R1	R2	R3			
0.02	0.021	0.018	0.017	0.019 ± 0.001	93.33	94.44
0.05	0.042	0.054	0.043	0.046 ± 0.002	92.67	
0.10	0.096	0.097	0.099	0.097 ± 0.010	97.33

The variation of active principles in *P. pinnata* depends on various factors, viz., age of the tree, location, season, soil and edaphic factors, etc. (Liu et al., 2015). This may lead to the variation in the performance of *P. pinnata* formulation against targeted pests. There are lots of variations in the behavior, physiology and genetic makeup of insects even if they belong to the same species (Ranjan et al., 2011). In addition to *karanjin*, the presence of other flavonoids and phenolic constituents in *P. pinnata* seed extract (Purkait et al., 2019) might increase the insecticidal and larvicidal activity of microcapsule formulation.

Mass fragmentation pattern of the chromatographic peak (RT = 21.94 min) was also comparable with the earlier reports (Patel and Trivedi, 2015). The precursor ion of *karanjin* at m/z 291 [C_18_H_**11**_O_4_]^+^produced the daughter mass ions at 263, 221, 179, 160, 132, 89 and 77 (Figure 2B). Previously, the qualifier product ions were reported as 221 [C_7_H_5_O_2_]^+^, 89 [C_7_H_5_]^+^ and 77 [C_6_H_6_]^+^(Patel and Trivedi, 2015). However, in this study, 179 [C_10_H_10_O_3_]^+^ was selected as a quantifier along with the other two 160 [C_10_H_8_O_2_]^+^ and 77 [C_6_H_6_]^+^ as qualifier ions (Table 1). Therefore, the appearance of the peak at RT 21.94 min was confirmed due to the occurrence of *karanjin* in PO.

## 4 Conclusion

This experiment describes the preparation, characterization, and evaluation of the insecticidal activity of botanicals in encapsulated systems. *P. pinnata* seed oil (5%) was successfully incorporated in polyuria microcapsules using interfacial polymerization technique in two-step processes with excellent physical and chemical properties. For the cross-linking of the monomers, a catalyst solution was used, which ensured a high degree of encapsulation (87.41%). *P. pinnata* microcapsule formulation (5%) presented high insecticidal activity against two important agricultural pests *A. gossypii* (71.8%) and *B. tabaci* (74.7 %). In addition to the sustained release, the technique of encapsulation may reduce the problems associated with microbial and photo-degradation, vaporization and increase the persistence of the bio-active ingredients (like the flavonoid *karanjin* and phenolic acids). The findings constitute an important contribution to the development of promising and eco-friendly advanced technology for crop protection in a sustainable manner.

## Declarations

### Author contribution statement

Aloke Purkait: Performed the experiments; Contributed reagents, materials, analysis tools or data; Analyzed and interpreted the data; Contributed reagents, materials, analysis tools or data; Wrote the paper.

Ayan Mukherjee; Dipak Kumar Hazra: Performed the experiments; Contributed reagents, materials, analysis tools or data.

Kusal Roy: Conceived and designed the experiments; Analyzed and interpreted the data; Wrote the paper.

Pabitra Kumar Biswas; Ramen Kumar Kole: Conceived and designed the experiments.

### Funding statement

This research did not receive any specific grant from funding agencies in the public, commercial, or not-for-profit sectors.

### Data availability statement

No data was used for the research described in the article.

### Declaration of interests statement

The authors declare no conflict of interest.

### Additional information

No additional information is available for this paper.
